# Evaluation of Compatibility between Beetle-Killed Lodgepole Pine (*Pinus Contorta* var*. Latifolia*) Wood with Portland Cement

**DOI:** 10.3390/ma3125311

**Published:** 2010-12-17

**Authors:** Sorin A. Pasca, Ian D. Hartley, Matthew E. Reid, Ronald W. Thring

**Affiliations:** 1 Ecosystem Science and Management Program, University of Northern British Columbia, 3333 University Way, Prince George, British Columbia, V2N 4Z9, Canada; E-Mail: spasca@unbc.ca; 2 Department of Physics, University of Northern British Columbia, 3333 University Way, Prince George, British Columbia, V2N 4Z9, Canada; E-Mail: mreid@unbc.ca; 3 Chemistry, Environmental Science and Environmental Engineering Programs, University of Northern British Columbia, 3333 University Way, Prince George, British Columbia, V2N 4Z9, Canada; E-Mail: thring@unbc.ca

**Keywords:** compatibility index, hydration, lodgepole pine, Portland cement, wood

## Abstract

The compatibility of wood from mountain pine beetle (*Dendroctonus ponderosa*) killed lodgepole pine (*Pinus contorta* var*. latifolia*) with Portland cement was investigated based on time-since-death as a quantitative estimator, and the presence of blue-stained sapwood, brown rot, or white rot as qualitative indicators. The exothermic behavior of cement hydration, maximum heat rate, time to reach this maximum, and total heat released within a 3.5–24 h interval were used for defining a new wood-cement compatibility index (CX). CX was developed and accounted for large discrepancies in assessing wood-cement compatibility compared to the previous methods. Using CX, no significant differences were found between fresh or beetle-killed wood with respect to the suitability for cement; except for the white rot samples which reached or exceeded the levels of incompatibility. An outstanding physicochemical behavior was also found for blue-stained sapwood and cement, producing significantly higher compatibility indices.

## 1. Introduction

Wood-cement composites are widely utilized in many countries for both interior and exterior applications because of their strength properties for building materials (e.g., siding, roofing, cladding, fencing and sub-flooring) and for acoustic properties such as in highway sound barriers [1]. These composites have unique advantages over other conventional materials, including durability, fire resistance, workability, and resistance to fungal and termite attack. The ability to use wood waste and the absence of petroleum-based binders and other additives, make wood-cement composites attractive, more environmentally friendly, cement composite. Organic material, including wood, inhibits the hardening of cement, and sugars, tannins, starches were among these compounds that adversely affected cement hydration [2]. Among North America wood species, lodgepole pine (*Pinus contorta* var. *latifolia* (Engelm.)) has the least inhibitory effect on cement hydration [3,4,5].

For more than a decade, the mountain pine beetle (*Dendroctonus ponderosa* [Hopkins]) infestation in British Columbia (BC) has affected millions of hectares of forest and resulted in hundreds of millions of cubic meters of dead, standing lodgepole pine trees. Storing timber prior to using it for wood-cement boards improves the compatibility between the wood particles and cement likely caused by a loss in some natural chemical inhibitors [6,7]. The amount of wood extractives in beetle-killed wood decreases with time-since-death (TSD) and enhances the beetle-killed wood’s suitability in the manufacture of wood-cement composites. In contrast, decayed wood was up to 50 times less compatible with Portland cement than heartwood [8]. The period of time before beetle-killed wood begins to deteriorate varies and depends on numerous factors, many of which may be impossible to readily predict [9]. Therefore, two main factors that affect the chemical interaction of beetle-killed wood with Portland cement are the improvement with the decrease in extractives amount and the reduction by decay through accumulation of many byproducts of the rotten wood.

The most common analytical method for assessing wood-cement compatibility is to measure the exothermic process of cement hydration. The approach is based on measuring the decrease of heat indicators during the hydration process as the inhibitor wood is added to cement paste. Maximum temperature, the time to reach this maximum value, the heat evolved over the first 24 h, and the maximum heat rate are among the most important calorimetric indicators of cement hydration. Initially, simple inhibitory indexes based on temperature were used [2,10], but more complex equations [3,4,11] were later developed to reduce the inconsistencies in classification [12].

Regardless of the assessment method, lodgepole pine was the most compatible North American wood species with cement [13]. Hofstrand *et al*. [3] calculated the inhibitory indexes for 9 softwood species and 12 hardwood species and found that lodgepole pine had a high level of suitability. Hachmi and Moslemi [14] determined the Ca-factor (CA) values for several wood species and obtained the highest value for lodgepole pine; lodgepole pine had the lowest extractive content (3.1%) among the species tested. Miller and Moslemi [5] found high values for hydration characteristics and tensile strength for lodgepole pine sapwood, but also reported significant differences between those values and values from heartwood. Since heartwood had a detrimental effect on both cement strength and exothermic behavior, the high degree of compatibility between lodgepole pine and cement was attributed to the large percentage of sapwood, probably due to the use of small diameter lodgepole pine trees that had a larger proportion of sapwood.

The percentage of extractives for lodgepole pine from BC varied from 1–2% in sapwood and 2–4% in heartwood [15] and total extractive amounts in fungal infested sapwood were about 60% lower than those in fresh sapwood. These levels could decline even further by the time trees reach the gray-stage, which occurs at four or more years since death. Extractive levels abruptly increase as live trees respond to beetle attack, but then decline to 1.2% in blue-stained sapwood [16]. Studies on southern pine report high levels of blue-stained sapwood-cement compatibility, expressed by an earlier rise in temperature [10] and a reduced setting time of the wood-cement mixtures [17]. However, the extractive content in heartwood was not significantly different between sound and infested wood [15]. In terms of wood-cement compatibility, the heartwood of pine species was a strong inhibitor of cement hydration [5], and therefore, highly unsuitable for the manufacture of wood-cement boards [18].

The objectives of this study were twofold: (1) to develop a new compatibility index; and (2) to assess the compatibility of beetle-killed lodgepole pine with Portland cement.

## 2. Results and Discussion

### 2.1. Development of New Compatibility Index (CX)

A new compatibility index (the cross compatibility index—CX) was developed that combined the affects of two other indices: CA [4] and CI [11]. The approach used for calculating CA was based on the comparison between wood-cement mixtures and neat cement paste with respect to the total heat released within 3.5–24 h interval. The intensity of reaction, expressed by the maximum heat equivalent rate and the equivalent time needed to reach that maximum heat was the key characteristic on which the CI calculation was based. However, accelerator agents could alter the CI, since the agents only reduce the time of setting of cement; and, therefore they artificially increase the value of the index, meant to be an overall compatibility assessor. For example, the majority of the samples in the current study using blue-stained sapwood produced CI > 100, but most of the hydration characteristics (maximum temperature, maximum heat rate, total heat) were evidently lower than those of neat cement.

**Figure 1:**
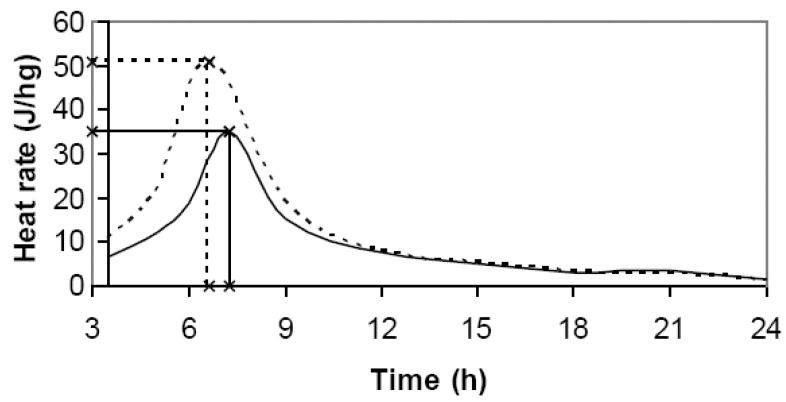
Heat rate versus time for wood-cement (solid curves) and neat-cement (dashed curves).

The cross compatibility index (CX) considered three elements in order to thoroughly cover the key aspects of hydration behavior: (1) maximum heat rate; (2) the time needed to reach that maximum; and (3) the total heat released within 3.5–24 h interval. An example of the calculation is presented in Figure 1. All of the calculations were performed on a “per gram of mixture” basis. The graph shows the determination of maximum heat rate, the time to reach that maximum, and the total heat (the area under heat rate curve) released within 3.5–24 h interval.

The heat rate was obtained by differentiating the heat equation given by Equation 1 [2,11]:

(1) $$HR_{i}=\frac{dH_{i}}{dt_{i}}=(C_{c}+C_{f})\left(\frac{d(T_{i}-T_{r})}{dt_{i}}+k(T_{i}-T_{r})\right)$$

where: *HR_i_* = heat rate at any instance i, (Jh^−1^); *H_i_* = total heat released by the mixture at any instant *i* between 3.5 and 24 h, (J); *C_c_* = total heat capacity of ingredients (cement + wood + water), (JK^−1^); *C_f_* = heat capacity of Dewar flask, (JK^−1^); *T_i_* = temperature of the mixture at any instant i, (K); *T_r_* = room temperature, (K); *k* = cooling rate constant, (h^−1^); *t_i_* = time at any instant *i*, (h).

The new index was calculated as the geometric mean, in this case, cubic root of there multiplied ratios: the maximum heat rate ratio, the total heat within 3.5–24 h interval ratio, and the time to reach the maximum heat inverse ratio, in Equation 2:

(2) $$CX\prime =\sqrt[{3}]{\frac{HR_{\max}}{HR_{\max}^{\prime }}\frac{H_{3.5-24}}{H_{3.5-24}^{\prime }}\frac{t_{\max}^{\prime }}{t_{\text{max}}}}$$

where: *HR_max_* = maximum heat rate of wood-cement mixture (Jh^−1^g^−1^); *HR´_max_* = maximum heat rate of neat cement paste (Jh^−1^g^−1^); *H_3.5–24_* = total heat released by wood-cement mixture in 3.5–24 h interval (J); *H´_3.5-24_* = total heat released by neat cement paste within 3.5–24 h interval (J); *t_max_* = time to reach maximum heat rate of wood-cement mixture (h); *t´_max_* = time to reach maximum heat rate of neat cement paste (h).

### 2.2. Assessment of Wood-Cement Compatibility

**Figure 2:**
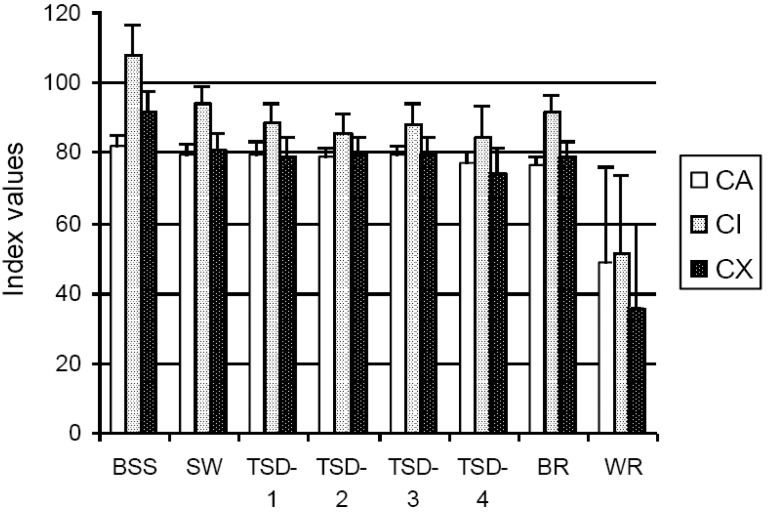
Wood-cement compatibility indexes (beetle-killed lodgepole pine and Portland cement).

Figure 2 presents the CA, CI, and CX means and standard deviations calculated for each of the eight groups. The results generated by using one-way ANOVA (Holm-Sidak method) gave the statistical grouping of the compatibility indexes means (Table 1). The values represent the means of ten observations, except for the white-rot group which contains only five observations.

**Table 1:** Statistical grouping of means of three wood-cement compatibility indices. Means with the same letter are not significant different at the 95 percent level.

Group	CA		CI		CX	
Blue-stained sapwood (BSS)	81.8	a	108.0	a	91.9	a
Sound wood (SW)	79.8	a	94.1	b	80.8	b
TSD-1	79.4	a	88.7	b	79.2	b
TSD-2	78.9	a	85.4	b	79.3	b
TSD-3	79.5	a	87.9	b	79.3	b
TSD-4	76.9	a	84.3	b	74.4	b
Brown rot (BR)	76.4	a	91.9	b	78.8	b
White rot (WR)	48.8	b	51.2	c	40.6	c

The ANOVA for CA showed significant difference between groups (*F_7,67_* = 12.123, *p* < 0.001). Tests using the Holm-Sidak methods were conducted to evaluate the pair-wise differences among the means identified only WR group being significant different than all the other groups at an overall significance level of 0.05. The CA induced a high degree of compatibility with cement, since they surpassed the proposed levels of compatibility defined by prior classifications. For instance, an upper CA = 68 was suggested the borderline between ‘moderate compatibility’ and ‘high compatibility’ and a lower CA = 28 as the threshold towards ‘incompatibility’ [14].

It was assumed that published CA indices were not calculated on a per-gram of mixture basis, but were calculated using the ratio of the heat released by two mixtures having different masses (305.5 g and 280 g, respectively). As a result, the indexes should be 8.35% larger than the values obtained in this study. For example, the mean CA of ‘sound’ group was 79.8, but it would become 86.5 based in former calculations. This value is in reasonable agreement with the CA = 85 obtained for lodgepole pine [4].

The mean value of 48.8 for the white rot group suggested a ‘moderate’ compatibility, although, the high standard deviation (27.1) might be a cause for concern. There were two samples at the upper limit of ‘incompatibility’ (CA = 16 and 28, respectively), meanwhile other two samples could be considered still compatible (CA = 75 and 76, respectively).

The differences among the groups were also significant (*F_7,67_* = 23.535, *p* < 0.001) when CI was taken into consideration as an indicator for wood-cement compatibility. All pair-wise multiple comparison procedure showed that both WR and BSS groups were significantly more compatible with cement than all the other groups. The results were in accordance with prior findings suggesting that mixtures containing blue-stained wood set earlier than mixtures containing non blue-stained wood [10]. This difference was attributed to the decrease in extractive content in the fungal colonized sapwood.

The mean CI for blue-stain sapwood was >100. This could be interpreted that blue-stained sapwood had a potential accelerator effect for setting of cement, since both effects of hydration, with a greater maximum heat evolution rate and especially a shorter equivalent time to reach that maximum, contributed to a higher CI than those of the neat cement. More research would be needed to understand the chemical composition of blue-stained sapwood and its interaction on cement hydration.

The CX showed similar statistically significant differences between groups (*F_7,67_* = 21.471, *p* < 0.001). The follow-up tests showed that the mean for the BSS group was higher than the means for all the other groups since almost all the times to reach the maximum heat rate of the blue-stained sapwood samples were shorter, even than the times characterizing neat cement paste. However, the maximum heat rates and the total heat released were below those of neat cement, so the CX means did not surpass the 100 mark.

There was no statistical difference among the ‘sound wood’ group and the TSD classes groups for any of the indexes. Taking into consideration the fact that the ‘sound wood’ group comprised both sound sapwood and sound heartwood, there was an assumption that the compatibility of beetle-killed wood would improve in real situations when the ‘high compatible’ blue-stained sapwood would be mixed with heartwood.

Beside the expected reduced suitability of white rot infested wood for cement mixtures, an important fact revealed by the analysis is that incipient ‘brown rot’ did not inhibit cement hydration, maintaining a high level of compatibility.

**Figure 3:**
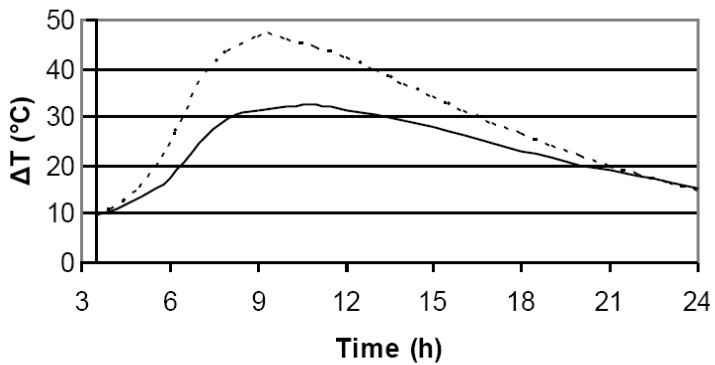
Hydration temperature for neat cement and wood-cement mixture. Values represent differences between the actual readings and the room temperature.

As illustrated in Figure 3, CX represented a combined effect of the CA and CI approaches. For example, low CA and CI merged into an even lower CX (“white rot” group), but the “exaggerated” CI (“blue-stained sapwood” group) was attenuated by a still higher CX but not exceeding 100.

The correlations among the three indexes were very high. A coefficient of determination *R²* of 0.787 was obtained when predicting CX by CA and a value *R²* = 0.876 was obtained predicting CX by CI. A multiple linear regression used for predicting CX function of both CA and CI produced a coefficient of determination *R²* = 0.932, which clearly demonstrated the predictive strength of the two indices (CA and CI) in generating the CX.

## 3. Experimental Section

The wood was collected from various sites in central British Columbia, where the initial stages of the beetle outbreak were traced back to the early 1990’s—Tweedsmuir Provincial Park (51°53’–53°49’N, 125°34’–127°22’W) and stands between Vanderhoof (54°02’N, 124°00’W) and Prince George (53°55’N, 122°47’W).

A classification to estimate time-since-death (TSD) based on external characteristics of the infested trees was used [19,20]. Four time-since-death (TSD 1–4) classes (Table 2) were considered along with two control groups: sound wood (SW) and blue-stained sapwood (BSS). The ‘sound’ samples were prepared by mixing sapwood and heartwood from uninfected lodgepole pine in an attempt to mimic the actual raw material used by the industry. The blue-stained lodgepole pine sapwood group was included to confirm prior studies showing an outstanding exothermic behavior of the stained wood-cement mixtures with different pine species [10,14]. The seventh group (BR) included specimens with visible indication of brown rot attack, although without affecting the physical integrity of the wood. Each group contained ten samples from ten different trees. Five discs identified as wood attacked by white rot fungi were considered as the eighth group (WR). Only the visual assessment was used in classifying the rotten samples. For consistency reasons, all the wood specimens but those from BSS and SW groups were heartwood.

**Table 2:** Description of the time-since-death classes for beetle-killed lodgepole pine. Modified from Thrower *et al.* [19] and Lewis *et al.* [20].

Time since death class	Estimated years since death	External tree appearance
TSD-1	1 year	green, yellowish or freshly red needles; no needles loss
TSD-2	2–3 years	red attack; slight needle loss
TSD-3	4–5 years	red attack; substantial needle loss
TSD-4	+6 years	gray attack; no needles, loss of fine branches

Normal Portland Cement (Type 10) was used in this study. The lodgepole pine wood was manually chopped into flakes and ground to pass through a size-20 mesh screen. Two types of mixtures were assessed in terms of hydration behavior: (1) Neat cement paste (200 g cement + 80 g water = 280 g); and, (2) wood-cement mixture (200 g cement + 15 g wood + 90.5 g water = 305.5 g).

The cement paste was mixed for 2 min, whereas the wood-cement mixtures were mixed for 5 min. Each sample was poured into a double-walled Dewar flask (Thermos^®^) which, in turn, was inserted into a fitted hollow built in a Styrofoam board. A SmartReader^®^ 6 Logger recorded the temperature in each container at 1 min intervals over a 24-h time period.

Calorimetric calculations such as heat rate, total heat within 3.5–24 h interval, and heat equivalence were based on the temperature differences (*ΔT*) between the actual recordings and the room temperature (21 ± 1 °C). An example of the differences between the plotted data from neat cement and wood-cement mixture is shown in Figure 3.

Two inhibitory indices: Ca-factor (CA) [4] and Compatibility Index (CI) [11] were calculated to assess the compatibility between wood and cement. Both methods were based on the exothermic chemical reaction of cement hydration and use the difference between neat cement paste and wood-cement mixtures.

## 4. Conclusions

Two approaches were employed in order to better evaluate most of the aspects of the exothermic process of wood-cement mixtures hydration, and a new index was proposed, combining the salient features of two previously proposed compatibility indexes. The sampling procedure was intended to comprehensively cover various characteristics of the beetle-killed wood; therefore, the results can be considered to have general relevance for an authentic evaluation of infested wood’s suitability for cement mixtures. The new proposed index CX is a reliable index of compatibility. Its calculation takes into consideration most of the exothermic characteristics of wood-cement mixtures hydration: maximum heat rate, time to reach that maximum heat rate, and total heat released during the chemical process. Therefore, CX is highly correlated with CA and CI, and especially with their combined interaction. CX accentuates wood-cement incompatibility when both CA and CI do so, but meanwhile, it diminishes the artificial “high” compatibility given especially through CI approach. The beetle-killed wood is at least as suitable as the ‘sound’ lodgepole pine for wood-cement composites. No evidence was found of limitations in terms of beetle-killed heartwood wood compatibility with cement in comparison with a control “sound” lodgepole pine wood. Moreover, the hydration characteristics were close enough to those belonging to neat cement paste, confirming that beetle-killed wood has a ‘high’ level of compatibility with Portland cement. Incipient decay produced by brown rot fungi does not significantly affect wood-cement compatibility. However, the level of suitability radically drops towards “incompatibility” by the time white rot fungi attacked the wood.
